# The impact of workplace factors on filing of workers’ compensation claims among nursing home workers

**DOI:** 10.1186/1471-2474-15-29

**Published:** 2014-01-29

**Authors:** Jin Qin, Alicia Kurowski, Rebecca Gore, Laura Punnett

**Affiliations:** 1 Department of Work Environment, University of Massachusetts Lowell, One University Avenue, 01854 Lowell, MA, USA

**Keywords:** Workers’ compensation, Healthcare worker, Work environment, Back pain, Under-reporting

## Abstract

**Background:**

Injuries reported to workers’ compensation (WC) system are often used to estimate incidence of health outcomes and evaluate interventions in musculoskeletal epidemiology studies. However, WC claims represent a relatively small subset of all musculoskeletal disorders among employed individuals, and perhaps not a representative subset. This study determined the influence of workplace and individual factors on filing of workers’ compensation claims by nursing home employees with back pain.

**Methods:**

Surveys were conducted in 18 skilled nursing facilities in four U.S. states. Self-administered questionnaires obtained information on demographic characteristics, working environment, and health behaviors/status. Employees who reported low back pain at least once in four questionnaire surveys were included. WC claims from the same facilities were obtained from the employer’s workers compensation insurer and matched by employee name. The dichotomous dependent variable was filing of back-related worker’s compensation claim. Association with predictors of interest, including pain severity, physical job demand, job strain, social support, schedule control, and safety climate, was assessed using multivariate regression modeling. Individual characteristics were tested as potential confounders.

**Results:**

Pain severity level was significantly associated with filing low-back related claims (odds ratio (OR) = 1.49, 95% CI = 1.18 – 1.87). Higher physical demands at work (OR = 1.07, 95% CI = 1.01 – 1.14) also increased the likelihood of claim filing. Higher job strain (OR = 0.83, 95% CI = 0.73 – 0.94), social support at work (OR = 0.90, 95% CI = 0.82 – 0.99), and education (OR = 0.79, 95% CI = 0.71 – 0.89) decreased the likelihood of claim filing.

**Conclusions:**

The results suggest that the WC system captured the most severe occupational injuries. Workplace factors had additional influence on workers’ decision to file claims, after adjusting for low back pain severity. Education was correlated with worker’s socioeconomic status; its influence on claim filing is difficult to interpret because of the possible mixed effects of working conditions, self-efficacy, and content knowledge.

## Background

Health care workers have a high risk of work-related musculoskeletal disorders and disabilities. The recorded incidence rate of musculoskeletal disorders among nursing aides, orderlies, and attendants was 239 per 10,000 full-time U.S. workers in 2011, the highest among all occupations reported [1]. The most common musculoskeletal disorder among health care workers is low back pain [2-4]. Reports of low back pain prevalence among nurses and other patient care workers range from 30% to 60% [4-7]. A recent study of hospital workers showed that prevalence of self-reported musculoskeletal symptoms in the past 3 months among nurses and patient care assistants was 74%, with 53% reporting pain in the low back [8]. Healthcare workers consistently rank among top occupations with disabling back conditions, primarily from patient handling activities [9-12]. Low back pain is a significant contributor to the nursing shortage [13].

Nursing assistants are the most frequently injured workers in health care [1] probably because they provide the majority of patient handling and moving tasks. Reported injuries to certified nursing assistants are three to four times that of registered nurses [14]. In 2009, the majority of injuries and illnesses (56%) among nursing aides involved patients, and 86% of those incidents were linked to overexertion [15].

Workers’ compensation (WC) claims is one of the important data sources used to estimate the scope of occupational injury and illness, prioritize resource allocation, and assess intervention usefulness. However, there are many filters before an occupational injury or illness is entered in any surveillance system [16]. Studies of compensation for work-related musculoskeletal disorders (WMSDs) have reported filing rates ranging from 6% to 46% in various populations with pain or diagnosed disorders in the low back or upper extremity [17-23]. Each of these studies reached the same conclusion: that many workers who may be eligible for benefits do not file WC claims. Capture-recapture analysis has demonstrated that workers’ compensation claims data, as a surveillance system, substantially underestimate the scope of occupational injuries and illnesses, including musculoskeletal disorders [24,25].

Factors that could affect workers’ decisions to file WC claims are multifaceted. Previously cited causes of underreporting include occupational factors (e.g. unionization, pressure from coworkers, corporate culture), injury and illness factors (e.g. severity, chronic/acute, failure to recognize work-relatedness), and personal factors (e.g. age, health insurance, fear of reprisal and job security, socioeconomic status) [26]. Instead of specific workplace factors, industry codes are often used as an imprecise indicator of workplace influence on workers’ reporting [27-29]. Few studies have quantitatively assessed a broad range of factors simultaneously. Further investigation of the impact of specific work environment characteristics on reporting to WC system is important to understand the issue of underreporting and utilization of the system.

The objective of this study is therefore to determine the influence of specific workplace characteristics on filing of WC claims among health care workers who reported low back pain. As part of a larger research effort to promote the mental and physical health of nursing home caregivers (“ProCare”), information was collected on work environment and employee characteristics, musculoskeletal symptoms, and workers’ compensation claims in a large chain of skilled nursing facilities (SNF’s) in the Eastern United States.

## Methods

### Data collection

This study included 18 nursing homes located in four states: Maine, Maryland, Massachusetts and Rhode Island. All 18 facilities were owned or managed by a single company. Starting from 2003, the company began to implement a “no-lift” safe resident handling program in its facilities. Four questionnaire surveys repeatedly measured exposure and health outcomes of all permanent full- and part- time employees. In 12 facilities, baseline surveys (F0) were collected during the week of the “no-lift” program implementation date, followed by surveys at 3 months (F1), one year (F2), and two years (F3) post-implementation. The other 6 facilities had already had resident handling equipment installed prior to the initiation of this research study, so their first surveys were conducted at least one year after implementation (F2). All surveys were collected in the period between May 2006 and October 2009.

Questionnaires were distributed at the nursing homes by our research team during scheduled break time over a two- to four-day period. Most employees completed and returned the questionnaires during their break time. For those who could not be met in person, such as third-shift and weekend employees, a pre-stamped, addressed return envelope was provided. Compensation of $20 was offered in exchange for each completed questionnaire returned. The study proposal was approved by the Institutional Review Board of the University of Massachusetts Lowell, and an informed consent form was obtained from each participant.

### Study design

The study population for these analyses is comprised of all employees who reported low back pain at least once in the four questionnaire surveys. Low back pain was identified if an employee checked “low back” in the question “During the past 3 months, have you had pain or aching in any of the areas shown in the diagram?” Participants rated their low back pain severity in five levels (none, mild, moderate, severe, and extreme).

The dichotomous dependent variable was filing of a worker’s compensation “First Report of Injury” (FRI) for a low back problem (1 = filed FRI, 0 = did not file). This variable represents the action of formal notification of a possible future claim without (yet) requesting or receiving any benefits. Most but not all of these FRI’s were accompanied by claims for medical and/or indemnity (lost-time wage replacement) costs. Information on the dependent variable came from the company’s worker’s compensation claim database for the study population between January 2003 and December 2010. A single WC insurance provider covered all 18 nursing homes. The WC claims associated with the back were extracted based on the body part code. When one individual filed multiple claims, only the first back claim was counted.

Workplace factors including job title, physical job demand, job strain, social support, schedule control, and safety climate were selected as the main predictors of interest. Job title was not included in the multivariable regression analyses because it was strongly correlated with other workplace variables. Low back pain severity was also included as an independent variable because it has been shown to be associated with WC reporting [23,30,31]. The self-administered questionnaire also collected detailed information on demographic characteristics, working environment, and health behaviors/status. To the extent possible, questions about workplace factors were derived from pre-existing, validated items and scales, including the Job Content Questionnaire (JCQ) [32], the SF-12 [33], safety climate [34] and schedule control factors [35].

Physical requirements at work were moving or lifting heavy loads, rapid and continuous physical activity and awkward postures. These three factors were summarized into one variable defined as physical job demand (range 5–20). The workplace variables were summarized to reduce collinearity among independent variables and to reduce their total number for regression modeling. Job strain (range1-16) was the ratio between JCQ items psychological job demand and decision latitude. High demand-control ratio means high job strain. Decision latitude was the sum of two items describing worker’s job decision authority and skill discretion. Social support (range 4–16) was the sum two items each for coworker support and supervisor support. Perceived workplace safety (range 1–4) was assessed with the sum of four items related to worker’s perception of workplace safety, adequate staffing, risk exposure, and management attitude towards health and safety. Schedule control (range 2–8) described how much the employee can control her/his work schedule.

### Data analysis

For each person in the study population, the highest pain severity level reported in any survey was used. If multiple surveys reported the same pain severity level, the earliest survey was used. Therefore, each person had one set of independent variables from one survey.

We tabulated the proportion of subjects with WC records, as well as the numbers of claims for medical and/or indemnity benefits. Workplace variable and pain severity were compared between nursing assistants and other job titles using Wilcoxon two-sided test (statistical significance at p = 0.05).

Associations between the main predictors and filing of low back WC claims were assessed with multivariate logistic regression to estimate odds ratios (OR) and 95% confidence intervals (CIs). Nineteen other variables were tested as potential confounders including 1) demographic and health factors: age, gender, race, education (years of schooling), BMI, marital status, chronic conditions (diabetes, hypertension, high cholesterol, Western Ontario McMaster Osteoarthritis Index), smoking, leisure activities (household and exercise), self-rated physical and mental health (SF-12 scales [33]), pain interference with work; 2) work-family factors: second job, child care, care for other dependent, work-family imbalance; 3) health beliefs: internal health locus of control [36], health self-efficacy [37]; and 4) state where the center was located (because workers’ compensation laws vary by state). If the effect estimates for any of the primary predictors changed by 10% or more after adding another variable to the regression model, the latter was determined to be a confounder and included in the final model. The statistical analyses were carried out with SAS statistical software (SAS Institute Inc., Cary, North Carolina, USA).

## Results

The F0 and F1 surveys were conducted at 12 SNF’s, the F2 surveys at 18 and the F3 at 15 SNF’s. Based on the employer’s personnel rosters, the total size of the workforce was 1282 in F0 and F1, 2187 in F2 and 1737 in F3. The average response rate across four questionnaire surveys was 74%, based on the numbers of usable surveys relative to employees on the complete workforce rosters. Out of the 2639 participants who returned at least one questionnaire survey, 1476 (55.9%) reported low back pain in the past 3 months at least once, so this represented the study base. The survey information from F0, F1, F2, and F3 was used for 366, 264, 529, and 314 people, respectively (three employees were excluded due to missing data).

The majority of the study population was female (90%) and under 55 years old (85%) (Table 1). Approximately 31%, 33%, and 36% of the participants were in the normal, overweight, and obese BMI category, respectively. Most of the participants had completed high school (41%) or college/professional education (53%). The study population consisted of 50% whites, 37% blacks, and 13% other races (including American Indians/Alaska native, Asian, mixed, and other). One hundred and seven people (8%) were self-identified as Hispanic. About half of the participants (733) were certified nursing assistants (CNA) or gerontological nursing assistant (GNA).

**Table 1:** Baseline sociodemographic characteristics of nursing home study population reported low back pain (n = 1476) and subjects who filed workers’ compensation claimsfrom 2003 to 2010 (n = 129)

	Number of survey respondents**	**% of total population**	**Number of claimants**	**% of total # of claim**	**Claim % in each row**
Age					
<=35	577	40.9	54	42.2	9.4
35-55	622	44.1	56	43.8	9.0
> = 55	213	15.1	18	14.1	8.5
Gender					
Female	1284	90.4	115	92.7	9.0
Male	137	9.6	9	7.3	6.6
BMI					
Normal (≤ 25 kg/m 2)	433	31.0	33	28.5	7.6
Overweight (25–30 kg/m 2)	459	32.9	39	33.6	8.5
Obese (≥ 30 kg/m 2)	505	36.2	44	37.9	8.7
Education					
Less than high school	12	0.8	1	0.8	8.3
High school	594	41.2	70	54.7	11.8
College/professional	760	52.7	56	43.8	7.4
Post-graduate	77	5.3	1	0.8	1.3
Race					
White	738	50.1	63	48.8	8.5
Black	544	36.9	40	31.0	7.4
Other	191	13.0	26	20.2	13.6
Hispanic					
Yes	107	8.4	17	14.4	15.9
No	1174	91.7	101	85.6	8.6
Job title*					
GNA/CNA	733	49.8	87	68.0	11.9
CMA	122	8.3	8	6.3	6.6
LPN	216	14.7	16	12.5	7.4
RN/RN manager	238	16.2	8	6.3	3.4
Other	164	11.1	9	7.0	5.5
State					
Massachusetts	255	17.3	36	27.9	14.1
Maryland	778	52.8	60	46.5	7.7
Maine	339	23.0	23	17.8	6.8
Rhode Island	101	6.9	10	7.8	9.9

*Job titles:certified nursing assistant (CNA), gerontological nursing assistant (GNA), licensed practical nurse (LPN), registered nurse/nurse manager (RN), certified medical assistant (CMA). “Other” includes rehabilitation, housekeeping, dietary, administration staff, personal care assistant, personal support specialist and social workers.

**Individual variables may sum to less than 1476 due to missing values.

A total of 129 survey participants (8.7%) filed low-back related workers’ compensation claims. Among the claims, 111 were stated to be caused by patient or material handling, 13 by slip, trips or falls, 2 by struck by objects, and 3 by other factors. Twenty-six employees filed two low back claims, two employees filed three claims, and another two filed four claims during the observation period. The numbers of claims before and after the implementation date of safe resident handling program were 32 and 97, respectively.

Of the 129 claims, 64 claims were filed before and 65 after the selected survey. The average time between the claim and the survey date was 641 (556) days for claims filed before the survey, and 561 (339) days for claims filed after the survey.

Out of 129 WC claims, 57 (44%) and 58 (45%) cases involved indemnity and medical only costs, respectively (Table 2). There were 50 (39%) claims that requested both medical and indemnity benefits, and 14 (11%) did not request any benefits. Among all the participants who filed claims, the education levels were lower for participants requesting indemnity benefits than those who did not (p-value = 0.05). In contrast, there was no difference in education between participants who filed claims requesting for medical benefits and those who did not (p-value = 0.95).

**Table 2:** Number of nursing home employees who filed back injury claims in each categories by job title

**Job title**	**Indemnity**	**Medical only**	**None**	**Total**
GNA/CNA/CMA	48 (50%)	40 (42%)	8 (8%)	96
LPN/RN	5 (21%)	13 (54%)	6 (25%)	24
Housekeeping/dietary/admin/other	4 (44%)	5 (56%)	0 (0%)	9
Total	57 (44%)	58 (45%)	14 (11%)	129

Job titles: certified nursing assistant (CNA), gerontological nursing assistant (GNA), certified medical assistant (CMA), licensed practical nurse (LPN), registered nurse/nurse manager (RN).

**Figure 1:**
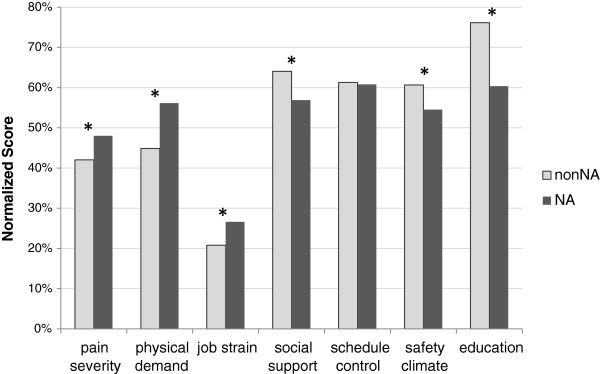
**Comparison of main predictors of interest between nursing assistants (NA) and participants in other job titles (nonNA).** normalized percentage score = (variable value – min)/(max – min) × 100%, *Wilcoxon test p-value < 0.05.

Nursing assistants, including CNA, GNA and CMA, were different from people in other job titles in terms of workplace factors, pain severity and education. Physical job demand, job strain and pain severity were higher among nursing assistants, and social support, safety climate, and education were lower among nursing assistants compared to people in other jobs (p ≤ 0.03) (Figure 1). Therefore, stratified analyses were performed for NA’s versus employees in other job titles.

The final model included six main predictors and two potential confounders of job strain – education and BMI. After including the latter in the model, lower job strain actually increased the probability of seeking WC benefits. When all participants were included in the model, pain severity level, physical demand, job strain, social support and education were all significantly associated with filing a low-back related WC claim (Table 3). Higher pain severity level and physical demand increased the likelihood of filing, while higher job strain, social support, and education decreased the likelihood of filing. Higher level of schedule control also tended to increase the probability of WC filing.

**Table 3:** Multivariate model of association between main predictors of interest and filing of workers’ compensation claims (number of claims = 129)

**Variable (range of values)**	**ALL**		**NA**		**nonNA**		Claim subset*	
	**(n = 1473)**		**(n = 856)**		**(n = 617)**		**(n = 1409)**	
	**OR**	**95% CI**	**OR**	**95% CI**	**OR**	**95% CI**	**OR**	**95% CI**
Pain severity (1–5)	1.49	1.18 – 1.87	1.63	1.25 – 2.12	1.03	0.63 – 1.70	1.34	1.00 – 1.80
Physical demand (5–20)	1.07	1.01 – 1.14	1.11	1.03 – 1.19	0.97	0.86 – 1.09	1.09	1.00 – 1.18
Job strain (1–16)	0.83	0.73 – 0.94	0.81	0.70 – 0.93	0.81	0.55 – 1.20	0.80	0.65 – 0.98
Social support (4–16)	0.90	0.82 – 0.99	0.92	0.83 – 1.02	0.81	0.66 – 1.00	0.90	0.79 – 1.03
Schedule control (2–8)	1.09	0.95 – 1.25	1.09	0.93 – 1.27	1.11	0.82 – 1.50	1.14	0.93 – 1.39
Safety climate (1–4)	1.04	0.67 – 1.61	1.20	0.73 – 1.96	0.78	0.28 – 2.15	1.16	0.66 – 2.03
Education (1–12)	0.79	0.71 – 0.89	0.84	0.71 – 1.00	0.78	0.64 – 0.95	0.80	0.68 – 0.93
BMI (11–57)	0.98	0.96 – 1.01	1.00	0.97 – 1.03	0.92	0.85 – 1.00	0.98	0.93 – 1.02

OR: odds ratio.

CI: confidence interval.

NA includes CNA, GNA and CMA, nonNA include other job titles listed in Table 1.

*Claim subset: only claims filed after the survey (number of claims = 65).

Being a nursing assistant (GNA, CNA or CMA) modified the relation between the main predictors and filing WC claims. For nursing assistants, pain severity, physical demand and job strain and education had a significant impact on claim filing (Table 3). For all other job titles, higher levels of social support, education and BMI decreased the likelihood of filing. The directions of associations in the stratified groups were the same as among all study participants. The results were similar when only claims filed after the survey were included in the model, except social support (OR 0.90; 95% CI 0.79 – 1.03).

## Discussion

This study examined the influence of workplace factors on filing of workers’ compensation claims among 1473 employees in 18 nursing homes. Only a small fraction (8.7%) of employees who reported low back pain in the self-administered questionnaires had filed workers’ compensation claims during the eight-year period that spanned both before and after the questionnaire surveys. Elevated pain severity level and physical work demand increased the likelihood of filing, while filing was less likely with higher social support, job strain, and education level. Nursing assistants were more likely to file claims if they had higher level of pain severity, higher physical job demand, and lower job strain. Claim filing was negatively correlated with social support and education level among employees in other job titles.

It is difficult to determine the work-relatedness of reported low back pain based on the information collected from the surveys. Undoubtedly it is possible that some of the back pain cases were not work-related. Due to lack of a more comprehensive surveillance system, WC database is still one of a few available data sources that could provide valuable information for studying work-related injuries and illnesses. This study highlighted that WC claims represented a small proportion of low back pain cases even in this very high-risk sector and pinpointed important factors associated with claim filing. Such information can be helpful for interpretation of results in future research using WC data to study work-related musculoskeletal disorders.

Workers’ compensation claims are known to represent only a fraction of all work-related disease [24]. There are barriers both before and after a worker decides to file a WC claim [16,26] that can filter out legitimate work-related conditions. These obstacles range from socioeconomic disincentive for an employee to inform the employer of a health problem, to physician lack of knowledge about or reluctance to follow the burdensome filing procedures. Further, many occupational illnesses, including back problems, are chronic and multifactorial, making it more difficult to ascertain etiology in any individual case. Documented reasons for underreporting of work-related musculoskeletal disorders by health care workers, in particular, include lack of time, peer pressure not to report, worker’s doubts about eligibility or severity, frustration with WC procedures, fear of reputation of being a complainer, income loss, career prospects, and workers’ belief that “injuries happen to most people with this occupation” and as such do not merit being reported [38,39].

The time and financial burdens of seeking medical attention, among other concerns [39], make it less likely that an affected worker would file a claim for a minor disorder, such as one that does not interfere with function at work or elsewhere. Our study confirmed prior reports that pain severity is a strong predictor of filing WC claims for WMSDs. Rosenman et al. [23] found that two significant correlates of WC filing for MSDs were degree of activity impairment and length of lost work time due to the condition. Another study [31] reported that filing was more likely when the MSD led to lost time or to surgery. Other associated factors in the two above-cited studies include working in physically demanding jobs such as manufacturing and/or a blue-collar occupation, length of employment, dissatisfaction with coworkers or with management support, and low socio-economic status (income or education). These findings agree with ours regarding not only MSD severity but also social support at work and socioeconomic status. The consistency is notable because the three studies used entirely different sampling approaches to define the study populations: reports of occupational disease from a single clinic [23], a survey of the general population in one state [31], and a survey of a single corporate workforce across several states (the present material).

High occupational physical demands, high job strain and low social support at work are known risk factors for MSDs in general and specifically in healthcare workers [40,41]. Our results showed that they also affect workers’ decisions to file WC claims. An investigation among workers in a single hospital showed the importance of working conditions in addition to socioeconomic status as predictors of WC claims and injuries in general [42], which is in accordance with our conclusions. Thus, controlling physical job demands to reasonable levels and improving social support at the workplace may not only help reduce the risks of the WMSDs but also decrease workers’ propensity to file claims.

Somewhat surprisingly, job strain had a negative correlation with claim filing. High job strain can result from low decision latitude and/or high psychological job demand. Similar to our finding, Keegel et al. [43] reported that workers at lower occupational skill level had more job strain but were underrepresented in WC claims. Morse et al. [31] observed higher reporting with lower decision latitude, unadjusted for other factors. In our data, the association between job strain and WC filing was strongly confounded by education and BMI index. Additional analysis of job strain suggested that its impact on claim filing mainly represented the control component (decision latitude) rather than the demand component.

Employees with lower education are more likely to have jobs that involve higher physical demand and therefore also have higher risks of developing WMSDs. It is noteworthy that in our analysis education was a significant predictor even after controlling for physical job demand and other workplace factors. This result suggests that education may also influence the decision about claim-filing through other mechanisms. We also found that the education level of workers who filed indemnity claims was lower than those who did not file indemnity claims, while there was no difference in education for claims with medical costs only. This supports the idea that workers in lower socioeconomic status have a higher dependence on WC benefits for lost wages due to missing work days, or that those with heavier work demands are more likely to need time off work due to low back pain.

It is also possible that employees with higher education have better health insurance and have more healthcare options in additional to WC than those with lower education. Several studies have shown that private or group health insurance affects the utilization of the WC system, and that many people choose instead to obtain care for WMSDs through health insurance [44,45]. We did not collect information on individual’s insurance coverage; however, we have been told that less than 50% of the nursing home workers in our sample enrolled in employer-offered group health insurance because of its cost.

There are several potential weaknesses in this study. The turnover rate of clinical staff in all 138 nursing facilities in the ProCare study was between 22% and 30% over the study period. Thus the healthy worker effect could not be ruled out, meaning that individuals who developed work-related back pain might have been more likely to leave employment than those who did not. Studies relying on data from respondents may suffer from recall bias, which can result in misclassification of the variables measured. The effect of recall bias depends on whether misclassification is systematically different between employees who filed claims and those who did not. For example, if the claim was filed after assessment of physical job exposures (survey administration), misclassification of physical job demands is likely to be independent of WC filing. If filing occurred before exposure assessment, it is possible that workload or work schedule may have been modified to lighter duty, leading to underestimation of etiologically relevant exposure. In addition, after the implementation of the “no-lift” safe resident handling program, work conditions were likely to be improved (e.g. physical job demand decreased). In all these cases, the resulting bias would be towards the null value. On the other hand, psychosocial conditions assessed after claim filing might in fact have worsened as a consequence of the claim being filed, such as from co-worker resentment or supervisor/management reprisal. To understand these issues, the final model was performed excluding claim cases filed before completing the survey (Table 3). The results were remarkably similar to those including all 129 claims filed either before or after the surveys, suggesting that the sequence of the claim and survey did not affect the associations observed in this study.

This study examined employees in 18 SNF’s owned and/or managed by a single company. Using this unique study population has advantages and limitations. On one hand, these nursing homes are similar in terms of organizational factors including occupational safety and health policy and programs, including resident handling equipment, training and practice; therefore the potential confounding effect from such group-level factors were minimized. On the other hand, the findings of this study among nursing home workers may not imply the same effect in other populations, even other healthcare workers. For instance, a higher proportion of injuries was reported by these nursing home employees than has been found in hospital settings [39,46]; workers in places that lack proper equipment to move and handle patients were less likely to report, and people trained in the use of equipment were more likely to report work-related injuries [38]. Further, union membership makes an individual worker more likely to file WC claims [28] but none of the nursing homes that we surveyed was unionized.

## Conclusions

Using a study population of employees in skilled nursing facilities, we showed that only a fraction of health care workers sought benefits through workers’ compensation despite high prevalence of musculoskeletal symptoms. A quantitative analysis shed light on how work environment characteristics affect workers propensity to utilize the WC system. Only the most severe cases of WMSDs were reported to the WC system. Workplace factors, including physical job demand, job strain and social support at work, had additional influence on workers’ claim filing after controlling for pain severity. Education also affected claim filing, which warrant future research. Workplace factors differed between nursing assistants and other job titles, and risk factors varied somewhat between job groups. Nursing assistants appeared to be more vulnerable to the effects of risk factors. The observed associations suggest that efforts to improve work environment health and safety will also reduce the likelihood of filing WC claims.

## Competing interests

The authors declare that they have no competing interests.

## Authors’ contributions

JQ: participated in study conception, performed data analysis and drafted the manuscript. AK: participated in data collection and preparation, and helped in data analysis. RG: participated in data management and preparation, and helped in data analysis. LP: conceived of the study, provided guidance in data analysis, and helped to draft the manuscript. All authors read and approved the final manuscript.

## Pre-publication history

The pre-publication history for this paper can be accessed here:

http://www.biomedcentral.com/1471-2474/15/29/prepub
